# A novel approach for portal system reconstruction in liver transplant patients with grade IV portal vein thrombosis: Case study and literature review

**DOI:** 10.3389/frtra.2022.922881

**Published:** 2022-10-20

**Authors:** Wei Qu, Zhi-Jun Zhu, Lin Wei

**Affiliations:** ^1^Beijing Friendship Hospital, Capital Medical University, Beijing, China; ^2^National Clinical Research Center for Digestive Diseases, Beijing Friendship Hospital, Beijing, China

**Keywords:** PVT, liver transplantation, Pull-out technique, double-approach procedure, portal hypertension

## Abstract

**Background:**

Portal vein thrombosis is a common problem of end-stage liver disease in patients with portal hypertension and Yerdel grade IV thrombosis may be a contraindication for liver transplantation. Advances in surgical technique have indicated the feasibility of liver transplantation with PVT such as Reno-portal anastomosis, cavo-portal hemitransposition, but low graft portal blood perfusion and regional portal hypertension were the limitations.

**Methods:**

We introduce a new approach for portal system reconstruction in a patient underwent liver transplantation: A 28-year-old male was diagnosed with Budd-Chari syndrome and portal hypertension with grade IV portal vein thrombosis.

**Results:**

The “Pull-out” technique was applicated for thrombectomy, which can aid in exposing the superior mesenteric vein and portal vein branches and reducing technical difficulties associated with the identification and dissociation of surrounding anatomical structures. To collect sufficient portal vein blood perfusion and avoid regional portal hypertension, the portal vein system was reconstructed through double-approach procedure: reno-portal anastomosis combined with portal-portal anastomosis.

**Conclusion:**

Based on a precision preoperative evaluation, application of the Pull-out technique and double-approach procedure may be an effective method of thrombectomy especially in cases of grade IV portal vein thrombosis.

## Introduction

Since the first successful liver transplantation by Starzl et al. in 1967, liver transplantation has become the standard therapy for many liver diseases, mainly chronic liver disease (1). Portal vein thrombosis (PVT) is a common problem in patients diagnosed of end-stage liver disease and portal hypertension. Doppler ultrasonography and magnetic resonance or computer tomography are the major diagnostic tools used for precision evaluation of PVT prior to liver transplantation. PVT incidence at the time of liver transplantation varies from 2.1–26% in different published clinical observations, and the risk of surgical complications increased in some severe cases receiving liver transplantation. Portal vein thrombosis poses an extremely difficult problem in cirrhotic patients who are in need of a liver transplant. The presence of PVT is associated with more technically difficult liver transplant and in certain cases can be a contraindication to liver transplant (2). For example, patients classified as Yerdel grade IV were considered as an relative contraindication for liver transplantation (3–5) as shown Table 1.

**Table 1:** PVT grading in liver transplantation according to the extent of thrombosis as based on the Yerdel classification.

**Grade**	**Description**
Grade 1	Partially thrombosis of PV, involving thrombus confined to < 50% of the vessel lumen, with or without minimal extension to the SMV
Grade 2	>50% occlusion of the PV, including total occlusions, with or without minimal extension to the SMV
Grade 3	Complete thrombosis of both PV and proximal SMV, with patent distal SMV
Grade 4	Complete thrombosis of PV as well as proximal and distal SMV

**Figure 1:**
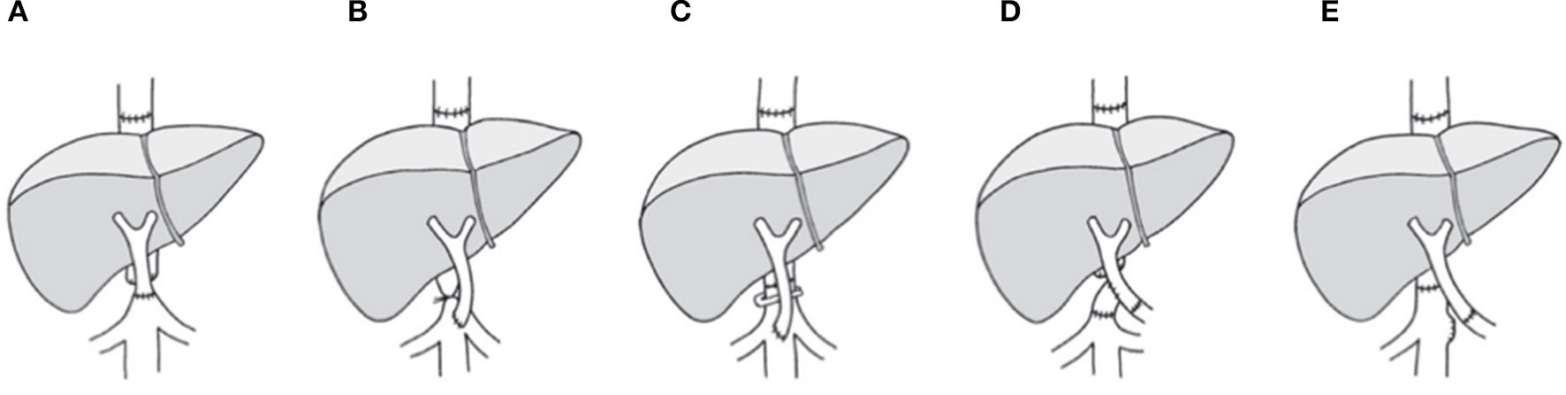
Approaches for portal flow reconstruction with systemic blood inflow as achieved with CPHT or RPA. **(A)** End-to-end cavoportal anastomosis. **(B)** Side-to-end cavoporal anastomosis between native inferior vena cava and portal vein graft with retro-hepatic caval vein constriction. **(C)** Side-to-end anastomosis between native inferior vena cava and portal vein graft preserving the retrohepatic caval flow by calibration of the retrohepatic caval vein. **(D)** End-to-side cavoportal anastomosis using the donor iliac vein interposition as a graft distal from conventional portal vein anastomosis. **(E)** End-to-end anastomosis between native left renal vein and portal vein graft. PVT, portal vein thrombosis; LTx, liver transplantation.

Recent clinical practice with aggressive surgical techniques of liver transplantation indicated the feasibility of liver transplantation even in the presence of extended PVT. A number of surgical approaches have been developed to sustain graft viability in cases with PVT related insufficient portal flow (6–8). In specific, liver transplantation for patients with PVT is possible with the implementation of various surgical techniques and strategies, such as renoportal anastomosis (RPA), cavoportal hemitransposition (CPHT) and collateral shunt vascular anastomosis, as shown in Figure 1. Ideally, the technique applied must provide a sufficient portal flow perfusion to the graft, and prevent post-operative persistence of regional portal hypertension simultaneously (9). RPA and CPHT had such limited technical defects as mentioned by Robles R.

Here, we introduce a new approach for portal system reconstruction in a Yerdel grade **IV** liver transplant patient. The PVT was diagnosed based on the precision evaluation, with complete thrombosis of SMV and splenic vein, being considered as a relative contraindication for liver transplantation. The patient received an orthotropic liver transplantation from a deceased donation liver graft allocated by COTRS (China Organ Transplant Response System, COTRS).

## Patient and surgical procedure

### Patient

**Figure 2:**
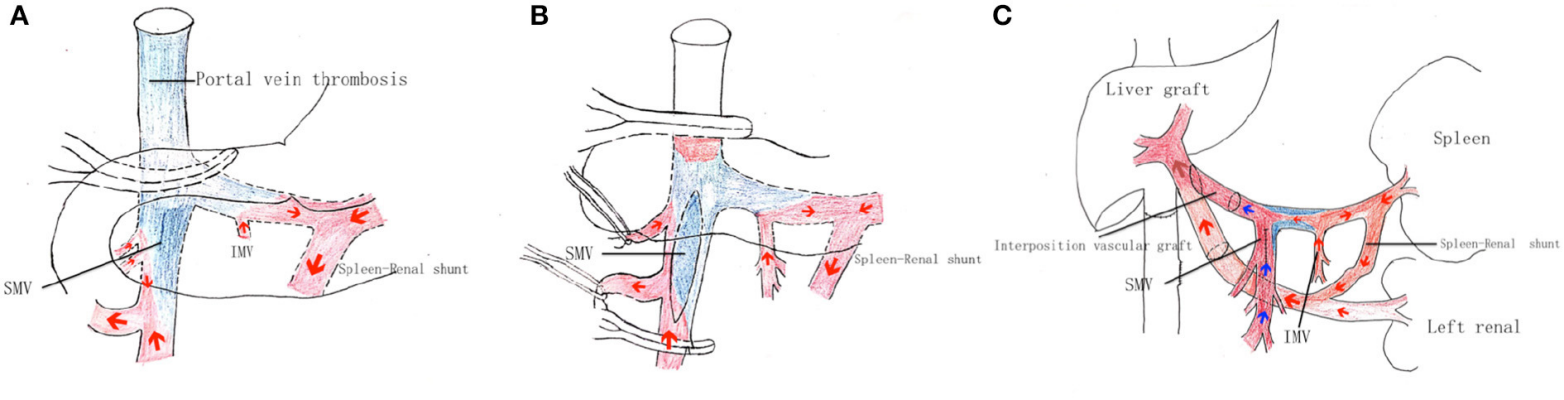
Surgical procedure of the portal system reconstruction. **(A)** First, the portal vein system was dissected and dissociated from the common trunk above the pancreas to the posterior region of the pancreas. The gastrocolic ligament was then dissected, exposing the pancreas, and revealing the mesenteric root from the anterior region of the pancreas. The mesenteric adipose tissue inferior to the pancreas was then dissected, exposing the distal portion of the superior mesenteric vein. In this way, dissection was performed at the inferior surface of the pancreas along the SMV branch to the SMV trunk, with this dissection and dissociation continued from the posterior to superior region of the pancreas to arrive at the confluence of the SPV and SMV. **(B)** The portal vein vessel wall was incised at the confluence of left and right portal branches and the thrombus was carefully dissociated from the upper surface by the stripper at the confluence of the SMV and SPV. The thrombus was partially dissected and removed, while blood flow was blocked using a portal vein clamp. Blood flow through the superior mesenteric vein branch was controlled by the trailing effect of the vascular suspension band, and the SMV was clamped longitudinally at the posterior direction of SMV branches using an auricular clamp. The anterior vessel wall of the SMV was incised to expose the thrombus and thrombus remnants were dissected and removed from the severed end. The anterior incision of the SMV was sutured and an evaluation of the PV blood perfusion was performed, but the portal vein blood flow was not sufficient. **(C)** The left renal vein (LRV) was fully exposed from the inferior hepatic vena cava to the distal inferior level of the left kidney vein. The LRV was isolated to a length of >2 cm, and the connected IVC wall was mobilized to more than two-thirds of its diameter. An end-to-end Reno portal anastomosis was then performed. The portal vein trunk of the recipient was anastomosed to the lateral side of the donor graft main portal vein with the donor iliac vein as an interposition vascular graft.

The patient was a 28-year-old male, diagnosed of Budd-Chari syndrome and portal hypertension with esophageal varices and recurrent bleeding. The region of portal vein thrombosis is shown as blue color in Figure 2. The blood flow collection from splenic vein and inferior mesenteric vein was accomplished backflow from the spontaneous spleen-renal shunt to the IVC through the left renal vein.

### Surgical procedure

Performance of the portal system reconstruction surgical procedure is described in detail below. The “Pull-out” technique was used to portal vein dissection in this liver transplant patient as shown in Figures 2A,B (10). This novel approach for portal flow reconstruction is illustrated in Figure 2C.

In order to confirm whether the blood perfusion of the portal vein system was sufficient, intraoperative Doppler Ultrasound examination was performed. When the blood flow of the recipient's portal vein was blocked, only with the RPA pathway retained, the flow velocity of the main portal vein of the transplanted liver was 21 cm/s. When the blood flow of the recipient's portal vein was opened, the flow velocity of the main portal vein of the transplanted liver was 35 cm/s, achieving satisfactory blood perfusion effect.

## Results

**Figure 3:**
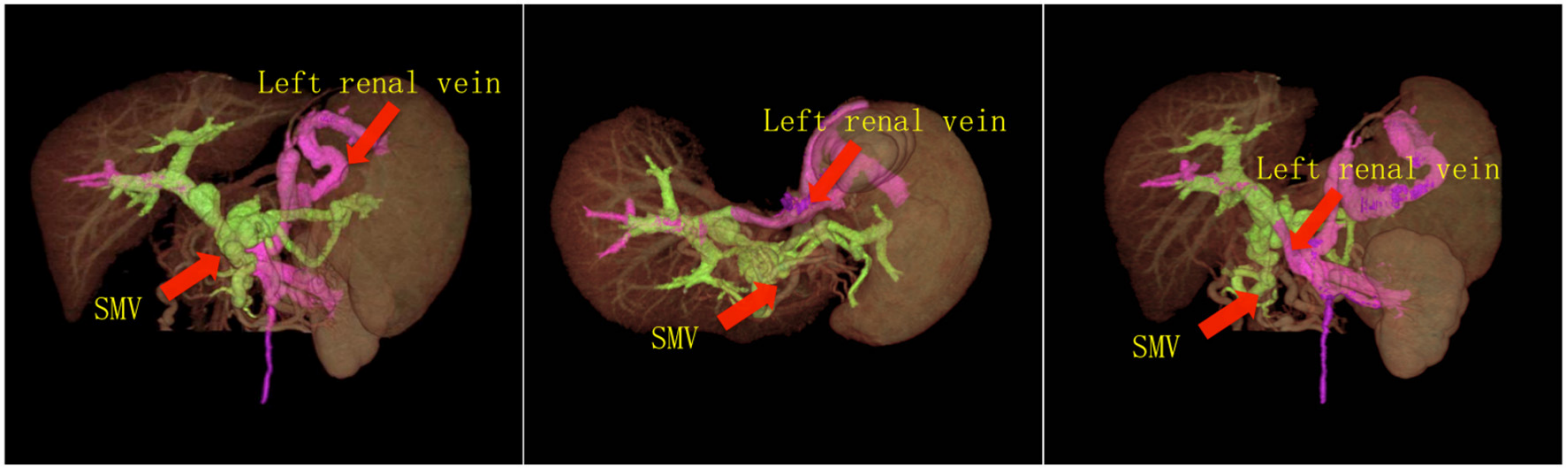
3-D CT images reconstruction of the portal system. The CT images showed that the portal vein perfusion was composed of blood flow from both PV and left renal vein, without regional portal hypertension.

Immunosuppression for the recipient consisted of tacrolimus, with a change to CSA at 2 weeks post liver transplantation, mycophenolate mofetil and methylprednisolone. To prevent recurrent portal vein thrombosis, heparin was administered within the first week post-operation. This prevention protocol continued with oral warfarin for 6 months. The patient recovered with no severe complications and normal liver functions were achieved. Mild renal dysfunction with peak serum creatinine 132 mmol/dl was diagnosed immediately after liver transplantation, and completed recovered 2 months post-transplantation. Doppler ultrasonography and computer tomography showed that the blood flow of hepatic artery, portal vein and hepatic vein were all normal, as shown in Figure 3. No severe postoperative complications such as thrombosis, bleeding, acute rejection, infection and biliary stricture ensued.

## Discussion

Completed thrombectomy in patients with grade **IV** PVT is a difficult surgical procedure. This procedure can result in massive bleeding and uncontrollable posterior vessel wall tear of the PV, which is a fatal event. Application of the Pull-out technique can aid in exposing the superior mesenteric vein and portal vein branches and reduce technical difficulties associated with the identification and dissociation of surrounding anatomical structures (11, 12). Thrombectomy performing from proximal and distal directional pathways, not only increases the safety of the surgical procedure, but also improves the effectiveness. However, this method does have some limitations. First, dissection of adipose tissue around the superior mesenteric vein can lead to damage of lymphatic channels in the root of mesentery. Even with extensive suturing to completely close the mesenteric tissue, a considerable amount of ascites or lymphatic leakage will be present after liver transplantation. Second, the extremely thin wall of the superior mesenteric vein precludes a complete removal of the thrombosis. Moreover, the smooth nature of the SMV endothelium can readily result in a re-thrombosis, requiring a protocol of heparin or warfarin anticoagulation treatment.

It should be noted that the main disadvantage of CPHT is the lack of decompression of the portal vein system. Symptoms of portal hypertension persist in some patients such as variceal bleeding and ascites, and some patients presenting with edema of the lower extremity or torso during the early post-operative course. However, these symptoms gradually diminish over a period of months after liver transplantation along with CPHT. In addition, ascites, renal dysfunction, and portal hypertension were also the main complications after RPA (13–15). If the portal vein reconstruction is limited to the portal system blood flow while discounting RPA and IMV reflux collection, hypertension will still persist in the SMV inflow region and the patient will be susceptible to symptoms of intestinal bleeding. If only an initial portal-to-portal anastomosis is performed, while disregarding blood collection of the SPV and IMV, regional splenic portal hypertension will persist with a low graft perfusion. To avoid this eventuality, we reconstructed the portal vein blood flow through two separate channels. This approach has the advantage of ensuring sufficient graft portal vein perfusion pressure and blood flow, while averting portal hypertension in the residual part of the reflux region. The major disadvantage is that resistance of left renal vein reflux would be higher than that of the inferior vena cava, which may lead to congestive injury of the kidney (16, 17). However, no severe complications such as kidney failure and proteinuria were observed in our present case.

With precision preoperative evaluation, the application of the Pull-out technique combined with double-approach procedure may improve the portal vein reconstruction in patients with grade **IV** portal vein thrombosis. The most important thing is the judgment of the anatomical morphology of collateral circulation of the portal vein system and the direction of collateral circulation blood flow. Not all the patients with grade **IV**PVT can benefit through this method, which is highly selective. With skilled surgical techniques, complex portal vein reconstruction can effectively supply sufficient portal vein perfusion and solve the persistent regional SMV or SPV hypertension.

## Data availability statement

The raw data supporting the conclusions of this article will be made available by the authors, without undue reservation.

## Ethics statement

Written informed consent was obtained from the individual(s) for the publication of any potentially identifiable images or data included in this article.

## Author contributions

WQ clinically managed the patients and wrote the manuscript. Z-JZ, WQ, and LW performed the surgical procedure. All authors read and approved the final version of the manuscript.

## Conflict of interest

The authors declare that the research was conducted in the absence of any commercial or financial relationships that could be construed as a potential conflict of interest.

## Publisher's note

All claims expressed in this article are solely those of the authors and do not necessarily represent those of their affiliated organizations, or those of the publisher, the editors and the reviewers. Any product that may be evaluated in this article, or claim that may be made by its manufacturer, is not guaranteed or endorsed by the publisher.

## Abbreviations

US: ultrasonography
SMV: superior mesenteric vein
ST: splanchnic tributary (coronary gastroepiploic vein)
MC: mesocaval shunt (spontaneous or surgically constructed)
SR: splenorenal shunt (spontaneous or surgically constructed)
RPA: renoportal anastomosis
CPHT: cavoportal hemitransposition
CPHT E-E: end-toend cavoportal hemitransposition
CPHT S-E: side-to-end cavoportal hemitransposition
IVC: inferior vena cava.
